# Staining patterns of autoantibodies in indirect immunofluorescence for autoimmune diseases of the digestive system: morphological characteristics and clinical significance

**DOI:** 10.3389/fimmu.2026.1796837

**Published:** 2026-07-15

**Authors:** Xiuying Shi, Xu Han, Yusi Liu, Min Zhao

**Affiliations:** 1 National Clinical Research Center for Medical Auxiliary Technology(Laboratory Medicine), Department of Laboratory Medicine, The First Hospital of China Medical University, Shenyang, China; 2 Department of Pathology, The First Hospital of China Medical University, Shenyang, China

**Keywords:** autoantibodies, autoimmune diseases, digestive system, IIF, morphological characteristics, staining patterns

## Abstract

Autoantibody detection is integral to the laboratory diagnosis of autoimmune diseases (AIDs). Notwithstanding the advent of a multiplicity of quantitative alternative methodologies, indirect immunofluorescence (IIF) remains the gold standard for the initial screening of many autoantibodies. Notably, autoimmune diseases of the digestive system present the widest variety of target autoantibodies and the most intricate fluorescence patterns, rendering them exceptionally contingent upon IIF testing. This article endeavors to furnish an exposition of autoantibodies germane to digestive system AIDs that necessitate IIF detection, meticulously delineating their fluorescent morphological characteristics and clinical significance. Given the complexity in interpreting IIF results for these specific autoantibodies, we will analyze fluorescent localization patterns based on tissue architecture, whilst disambiguating them from common confounding artifacts encountered in clinical practice. This study marks a dedicated attempt to differentiate among several antibody groups with overlapping fluorescence profiles, namely, anti-smooth muscle antibodies (ASMA), anti-elastin antibodies, and anti-reticulin antibodies (ARA); as well as antimitochondrial antibodies (AMA), anti-parietal cell antibodies (PCA), and heterophile antibodies (HA). The ultimate objective is to augment the pattern recognition and interpretive acumen of both laboratory professionals and clinicians, thereby maximizing the benefit of IIF testing in patients with suspected conditions.

## 1 Introduction

Autoantibodies are intrinsically linked to autoimmune diseases (AIDs), serving as indispensable biomarkers that facilitate the diagnosis of a panoply of autoimmune conditions (1–4). In the detection of autoantibodies associated with digestive, rheumatoid, and cutaneous autoimmune diseases, indirect immunofluorescence (IIF) remains a widely used method. Among these, autoimmune diseases of the digestive system are distinguished by the most labyrinthine spectrum in both types and patterns of target autoantibodies, thereby mandating a profound reliance on IIF for their diagnosis. AIDs of the digestive system principally encompass autoimmune liver diseases, which include autoimmune hepatitis (AIH), primary biliary cholangitis (PBC), and primary sclerosing cholangitis (PSC); along with gastrointestinal autoimmune conditions, such as autoimmune gastritis (AIG), ulcerative colitis (UC), Crohn’s disease (CD), and celiac disease (gluten-sensitive enteropathy) (5–7). Although alternative methodologies, including chemiluminescence immunoassay (CLIA) and enzyme-linked immunosorbent assay (ELISA), have been developed to replace IIF for certain autoantibodies, the detection of autoantibodies associated with digestive system AIDs continues to rely predominantly on IIF according to current guidelines, particularly for autoantibodies, namely antinuclear antibodies (ANA), anti-smooth muscle antibodies (ASMA), anti-liver-kidney microsomal antibodies (LKM), and antimitochondrial antibodies (AMA) (6–9). This study pioneers a differentiation between analogous fluorescence patterns of antibodies, notably distinguishing ASMA from anti-elastin and anti-reticulin antibodies, as well as AMA from anti-parietal cell antibodies and anti-brush border antibodies of the renal proximal tubular epithelium, thereby enhancing diagnostic precision for digestive system AIDs.

## 2 Methods

### 2.1 Literature review and article selection

A literature review was performed using the PubMed database (1975–2025) to examine autoantibodies detected by indirect immunofluorescence in autoimmune diseases of the digestive system. The search terms included: autoantibodies, fluorescent patterns, morphological characteristics, indirect immunofluorescence, fluorescent staining, gastrointestinal autoimmune diseases, autoimmune liver disease, clinical significance, anti-smooth muscle antibody (ASMA), anti-elastin antibody, anti-reticulin antibody (ARA), anti-mitochondrial antibody (AMA), anti-parietal cell antibody (PCA), heterophile antibody (HA), antinuclear antibody (ANA), anti-liver-kidney microsomal antibody (LKM), anti-liver cytosol type 1 antibody (LC1), anti-liver cell membrane antibody (LMA), anti-liver specific membrane lipoprotein antibody (LSP), and anti-neutrophil cytoplasmic antibody (ANCA). Articles published online, in print, or in press across all years were included in the analysis. Articles in English, or non-English articles with an English abstract, were considered eligible. Reference lists of the initially identified articles were additionally screened to identify further eligible publications.

## 3 Fluorescent patterns of autoantibodies associated with autoimmune liver disease

Autoantibodies associated with autoimmune liver diseases (AILD) primarily include anti-smooth muscle antibody (ASMA), a specific autoantibody for type 1 autoimmune hepatitis (AIH-1), antinuclear antibody (ANA), a sensitive autoantibody for AIH-1, anti-liver-kidney microsomal antibody (LKM) and anti-liver cytosol type 1 antibody (LC1), both associated with type 2 autoimmune hepatitis (AIH-2), antimitochondrial antibody (AMA), a specific autoantibody for PBC, along with anti-soluble liver antigen/liver-pancreas antibody (anti-SLA/LP antibody), anti-liver cell membrane antibody (LMA) and anti-liver specific membrane lipoprotein antibody (LSP) (9–11). Notably, anti-SLA/LP antibody is devoid of a characteristic fluorescent pattern, whereas LMA and LSP are organ (hepatic)-specific (12–14). IIF remains the benchmark assay for screening ANA, ASMA, LKM1, and AMA antibodies (9, 15, 16). The morphological characteristics and clinical significance of autoantibodies for autoimmune liver diseases are summarized in **Table 1**.

**Table 1:** The morphological characteristics and clinical significance of autoantibodies for autoimmune liver diseases.

The type of autoantibodies	Morphological characteristics				Related autoimmune diseases of the digestive system	Ref.
	Rat stomach substrates	Rat kidney substrates	Liver substrates	HEp2-cells		
ASMA	The staining of the interglandular contractile fibers within the mucosa lamina propria, in conjunction with the smooth muscle cells of the muscularis mucosae and muscularis propria.	The staining of arterial vessels, glomerular mesangial region, and intracellular fibrils in renal tubules.	Y-shaped fluorescent staining indicating the bile canaliculi surrounding hepatocytes (monkey liver substrate).	Cytoplasmic fibrillar linear pattern (AC-15).	Type 1 autoimmune hepatitis (AIH-1).	(8, 9, 15–18)
ARA	The staining of peritubular fibers, vascular endothelium and perivascular fibers.	The staining surrounding blood vessels, renal tubules or glomeruli.	The nodular fluorescence on the connective tissue surrounding the portal venule, linear fluorescence of the hepatic sinusoid, with “hair-like” extensions into the hepatic parenchyma (rat liver substrate).	–	Celiac disease.	(21–23, 66)
AMA	The staining of parietal cells.	The cytoplasmic speckled fluorescence on tubular epithelial cells.	The cytoplasmic speckled fluorescence on hepatocytes.	Cytoplasmic reticular pattern (AC-21)	Primary biliary cholangitis (PBC).	(8, 9, 15, 16, 38, 39)
PCA	The staining of parietal cells.	–	–	–	Autoimmune gastritis (AIG) and pernicious anemia (PA).	(40, 41, 43, 44)
HA	The staining of parietal cells.	The staining of the brush border in proximal renal tubular epithelium.	–	–	Unclear.	(53–55)
ANA	–	–	The interpretation of certain patterns (dense fine speckled, nuclear membrane, mitochondrial-like, etc.).	AC1-AC31.	AIH-1, PBC, etc.	(8, 9, 15, 16, 25–28).
LKM	–	An overall “half-positive, half-negative” pattern.	Cytoplasmic fine speckled to homogeneous fluorescence(rat liver substrate).	–	Type 2 autoimmune hepatitis (AIH-2).	(11, 31).
LC-1	–	–	The cytoplasmic staining of hepatocytes with a weakening of the staining around the central vein (rat liver substrate).	–	AIH-2.	(8, 9, 15, 16)
LMA	–	–	The membranous fluorescence on the hepatocyte membrane and reticular fluorescence on liver substrate.	–	Chronic active liver diseases.	(13, 14)
LSP	–	–	The granular cytoplasmic fluorescence on monkey liver substrate but demonstrates minimal fluorescence on rat hepatocytes.	–	An organ (hepatic)-specific autoantibody.	(13, 14)

ASMA, anti-smooth muscle antibody; ARA, anti-reticulin antibody; AMA, anti-mitochondrial antibody; PCA, anti-parietal cell antibody; HA, heterophile antibody; ANA, antinuclear antibody; LKM, anti-liver-kidney microsomal antibody; LC-1, anti-liver cytosol type 1 antibody; LMA, anti-liver membrane antibody; LSP, anti-liver-specific membrane lipoprotein antibody; -, negative or no specific fluorescence.

### 3.1 Anti-smooth muscle antibody

According to current guidelines, IIF of ASMA should be performed using freshly frozen triple rodent (typically rat) tissue as substrates, with rat stomach tissue serving as the standard control substrate for this assay (8, 9, 15, 16). Fresh-frozen rodent kidney and liver tissues, along with HEp-2 cell line, can serve as adjunctive substrates for differential diagnosis (8, 9, 17, 18).

The initial identification of ASMA is conducted on rat stomach tissue substrates. ASMA positivity is characterized by discrete staining of the interglandular contractile fibers within the mucosa lamina propria, in conjunction with the smooth muscle cells of the muscularis mucosae and muscularis propria (8, 9, 15–18). It is imperative to note that the fluorescence intensity should be relatively homogenous across all three aforementioned components. When fluorescent staining is observed exclusively in the gastric muscularis propria and muscularis mucosae without involvement of interglandular contractile fibers, this pattern should not be interpreted as ASMA positivity but rather classified as non-specific fluorescence. Furthermore, the presence of fluorescence in the submucosal vascular smooth muscle can serve as an auxiliary diagnostic criterion.

Clinically, differentiation of ASMA from anti-elastin antibodies and anti-reticulin antibodies (ARA) is necessary. Elastin is the primary component of elastic fibers, which are distributed basically in ligaments and vasculatures (19, 20). Elastin is located in ligaments and vasculatures. In contrast, fluorescence caused by ARA appears as fibrous fluorescent staining between gastric glands and a “honeycomb-like” fluorescence pattern on the smooth muscle region (21–23).

Collectively, on the mucosa lamina propria, ASMA and ARA share similar interglandular fluorescent pattern, whereas on the muscularis mucosae and muscularis propria, ASMA manifested as diffuse, intense positivity within the smooth muscle cells, while ARA tended to delineate the contours of the smooth muscle. In contrast to anti-elastin antibodies, which lacked specificity, ARA displayed a more characteristic honeycomb fluorescent pattern, however, supplementary methodologies are needed to distinguish these two due to overlapping fluorescent localization.

Following confirmation of ASMA positivity, three distinct subtypes, namely ASMA-V, ASMA-VG, and ASMA-VGT, can be designated based on IIF patterns observed on rodent kidney substrate (8, 9, 15–17). ASMA-V is characterized by the fluorescent staining limited to the smooth muscle fibers within arterial vessels, with no specific fluorescence observed on glomeruli or renal tubule (8, 9, 15–17). ASMA-VG shows the fluorescent staining of both arterial vessels and glomerular mesangial region, with no significant fluorescence observed on renal tubules (8, 9, 15–17). ASMA-VGT exhibits the fluorescent staining of all three structures: arterial vessels, glomerular mesangial region, and intracellular fibrils in renal tubules (8, 9, 15–17).

The ASMA-V subtype exhibits limited disease specificity and has been identified in several hepatic disorders, whereas the ASMA-VG and VGT subtypes, which demonstrate reactivity to F-actin, possess higher specificity for AIH (8, 9, 15–17). Therefore, it is recommended that additional remarks regarding the ASMA subtypes should be included in positive reports. Anti-elastin antibody stains vascular walls rather specifically without inducing signals in glomeruli or renal tubules, whereas ARA can simultaneously outline the vasculatures, the glomeruli, and the renal tubules. Unlike the characteristic transmural fluorescence of the vascular muscular layer caused by ASMA, both anti-elastin antibody and ARA generate linear fibrous fluorescence on vascular walls.

Fluorescence on liver substrate provides strong supporting evidence for ASMA diagnosis as well. On rat or monkey liver substrate, ASMA generates Y-shaped fluorescent staining indicating the bile canaliculi surrounding hepatocytes (8, 9, 15, 16). It is important to note that this pattern is not universal in ASMA-positive patients; however, prominent fluorescent staining of the vascular muscular layer is generally observable. This indirectly confirms that prominent vascular muscular fluorescence is a fundamental but non-specific feature of ASMA. Meanwhile, neither anti-elastin antibody nor ARA produce Y-shaped fluorescent staining on rat liver substrate. Anti-elastin antibody causes distinct linear fibrous fluorescence on the vascular wall (19, 20), whereas ARA induces nodular fluorescence on the connective tissue surrounding the portal venule, linear fluorescence of the hepatic sinusoid, with “hair-like” extensions into the hepatic parenchyma (21–23). High fluorescence intensity of ARA may create a visual illusion resembling the transmural fluorescence induced by ASMA, hence careful differentiation between these patterns is essential, and gradient dilution should be performed when necessary to ensure accurate interpretation.

On HEp-2 cells, ASMA typically demonstrates a cytoplasmic fibrillar linear pattern (AC-15), primarily involving filamentous actin (anti-F-actin, which is present in 80% of AIH patients with ASMA-VG/VGT patterns) and is further characterized by “firewood-like” bundled fibers (8, 9, 15, 16). Additionally, cytoplasmic fibrillar filamentous pattern (AC-16) may be present in ASMA-positive patients, but with low specificity (24). It is important to note that non-specific cytoplasmic fiber fluorescence may coexist with actin-type fiber fluorescence.

The diagnostic algorithm for ASMA detection involves a systematic approach across multiple substrate platforms. Initially, ASMA positivity is established by demonstrating distinct fluorescence with consistent intensity across the muscular layer, muscularis mucosae, and inter-glandular contractile fibers of rodent stomach tissue. Subsequently, ASMA subtypes (V/VG/VGT) are differentiated based on the presence or absence of fluorescent staining in the vascular muscular layer (V), glomerular mesangial cells (G), and intracytoplasmic fibrils of renal tubular cells (T) on rodent kidney tissue substrate. Supporting evidence is provided by Y-shaped fluorescence of the bile canaliculi surrounding hepatocytes, though this finding is considered optimal but not essential for diagnosis. Additionally, cytoskeletal stress fiber staining on HEp-2 cells, particularly the actin-type cytoplasmic fiber pattern, contributes to the comprehensive assessment of ASMA positivity. Notably, no specific fluorescence is observed on other tissue substrates.

### 3.2 Antinuclear antibody

ANA is frequently detected in multiple autoimmune disorders, including Systemic Lupus Erythematosus (SLE), Sjögren’s Syndrome (SS), and Progressive Systemic Sclerosis (PSS), etc (25–27). The interpretation of ANA patterns is essential for differentiating digestive system autoimmune diseases, with IIF remaining the reference method for ANA detection (8, 9, 15, 16, 25–27). Current guidelines recommend HEp-2 cells as the primary substrate for IIF screening, with monkey liver tissue serving as an adjunct substrate for pattern differentiation (8, 9, 15, 16, 25–27). Methods such as ELISA, WB, and CLIA are employed to confirm target antigens. Given that numerous ANA target antigens remain unidentified, IIF will continue to serve as an essential and irreplaceable technique for ANA detection in the foreseeable future.

According to international consensus guidelines, ANA staining patterns are classified into distinct categories: Nuclear patterns include homogeneous (AC-1), speckled subtypes [dense fine speckled (AC-2), fine speckled (AC-4), large/coarse speckled (AC-5), Topo I-like (AC-29), myriad discrete speckled nuclear pattern (AC-31)], centromere/CENP (AC-3), dot patterns [multiple nuclear dots (AC-6), few nuclear dots (AC-7)], nucleolar patterns [homogeneous nucleolar (AC-8), clumpy nucleolar (AC-9), punctate nucleolar (AC-10)], and nuclear membrane patterns [smooth nuclear envelope (AC-11), punctate nuclear envelope (AC-12)]. Nuclear polymorphic patterns include Anti-PCNA (AC-13) and CENP-F-like (AC-14). Cytoskeletal patterns comprise cytoplasmic fibrillar linear/actin-like (AC-15), cytoplasmic fibrillar filamentous (AC-16) including vimentin-like, keratin-like, and tubulin-like variants, and cytoplasmic fibrillar segmental/vinculin-like (AC-17). Cytoplasmic speckled patterns include cytoplasmic discrete dots/GW body-like (AC-18), cytoplasmic dense speckled (AC-19), and cytoplasmic fine speckled (AC-20). Other cytoplasmic patterns encompass cytoplasmic reticular/AMA (AC-21), cytoplasmic polar/Golgi-like (AC-22), and cytoplasmic rods and rings (AC-23). Mitotic nuclear patterns include centrosome (AC-24), spindle fibers/non-NuMA (AC-25), NuMA-like (AC-26), intercellular bridge (AC-27), mitotic chromosomal (AC-28) and fine-speckled nuclear with mitotic plate (AC-30) (8, 9, 15, 16, 25–27).

While most ANA staining patterns can be directly identified on HEp-2 cells, the interpretation of certain patterns (dense fine speckled, nuclear membrane, mitochondrial-like, etc.) demonstrates enhanced reliability when combined with fluorescence characteristics observed on monkey liver substrates.

Common ANA nuclear patterns associated with digestive system autoimmune diseases include nuclear homogeneous, nuclear speckled, nucleolar, centromere (CENP), and cytoskeletal patterns (28–30). Among these, ASMA (the specific autoantibody for AIH-1) is frequently associated with the cytoskeletal pattern in ANA, demonstrating a particularly strong correlation with the cytoplasmic fibrillar linear/actin-like pattern (AC-15) (8, 9, 27). The specific autoantibody in PBC patients is predominantly AMA, which corresponds to the cytoplasmic reticular (AC-21) ANA pattern on HEp-2 cells (10, 27, 28). Additionally, both nuclear dot and nuclear membrane patterns demonstrate significant associations with PBC; specifically, the multiple nuclear dots pattern (AC-6) and punctate nuclear envelope pattern (AC-12) exhibit high specificity for PBC (10, 27, 28). The multiple nuclear dot pattern corresponds to autoantibodies against soluble acidic phosphoprotein 100 (SP100), whereas the punctate nuclear envelope pattern corresponds to autoantibodies against transmembrane glycoprotein 210 (GP210) (10, 27, 28). Both autoantibodies demonstrate high specificity for PBC. Although ANA positivity is observed in PSC patients, no specific ANA staining pattern has been identified that definitively indicates PSC.

### 3.3 Anti-liver kidney microsome antibody

LKM1, LKM2, and LKM3. Anti-LKM1 antibodies are relatively common, while anti-LKM3 antibodies are rare; both are closely associated with AIH-2 (11, 31). Anti-LKM2 antibodies are associated with Drug-Induced Liver Injury (DILI) but not with AIH (32–34). LKM could be clarified by indirect immunofluorescence, but it is not possible to differentiate the LKM1 pattern from the LKM3 pattern. Rodent kidney tissue serves as the standard substrate for detecting anti-LKM1, with fluorescence patterns on liver substrate providing auxiliary diagnostic evidence (8, 9, 15, 16). Rat kidney and liver substrates are demonstrated here as examples.

On rat kidney substrate, anti-LKM1 antibodies are characterized by cytoplasmic speckled fluorescence in proximal renal tubular cells (with nuclear sparing), negative staining of distal renal tubular cells, resulting in an overall “half-positive, half-negative” pattern, accompanied by weak fluorescence in the glomeruli (8, 9, 15, 16). On rat liver substrate, LKM1 exhibits cytoplasmic fine speckled to homogeneous fluorescence. No specific fluorescence patterns are observed on other substrates. It is important to distinguish anti-LKM1 from AMA. On kidney substrate, AMA shows a speckled fluorescence pattern on the cytoplasm of both proximal and distal renal tubular epithelial cells, with weak fluorescence on glomeruli. On liver substrate, AMA induces speckled cytoplasmic fluorescence on hepatocytes.

Anti-LKM1 positivity is established by the presence of granular cytoplasmic fluorescence in proximal renal tubules, negative staining in distal renal tubules, and an overall “half-positive, half-negative” pattern on rat kidney substrate.

### 3.4 Anti-liver cytosol antibody type 1

Anti-LC1 represents an organ-specific autoantibody with low detection prevalence, serving as the sole detectable autoantibody in a subset of AIH-2 patients and demonstrating correlation with disease activity and progression (9, 35, 36). Detection of anti-LC1 is recommended using either ELISA or IIF on rat liver substrate (8, 9, 15, 16). For IIF detection of anti-LC1, the cytoplasmic staining of hepatocytes with a weakening of the staining around the central vein, resulting in an overall heterogeneous fluorescence intensity pattern; this phenomenon is absent on monkey liver substrate. The determination of anti-LC1 positivity should be based on the combined fluorescence patterns observed on both rat and monkey liver substrates. When anti-LC1 is present in isolation, the cytoplasm of monkey hepatocytes exhibits homogeneous fluorescent staining. However, anti-LC1 frequently coexists with anti-LKM1 (37). When both antibodies are present simultaneously, the fluorescence changes induced by anti-LC1 are masked by those caused by anti-LKM1. In such cases, identification using IIF alone becomes challenging, and alternative detection methods such as ELISA or Western blotting are recommended for differentiation.

In summary, isolated anti-LC1 positivity is characterized by significantly diminished cytoplasmic staining of hepatocytes surrounding the hepatic veins on rat liver substrates, demonstrating an overall fluorescence pattern of heterogeneous intensity.

### 3.5 Anti-soluble liver antigen/liver pancreas antibody, anti-liver membrane antibody, and anti-liver-specific membrane lipoprotein antibody

Anti-SLA/LP antibodies serve as valuable biomarkers for AIH-I diagnosis and assist in evaluating disease severity and prognosis (8, 9, 15, 16). Due to the absence of characteristic fluorescent staining patterns, IIF cannot be employed for anti-SLA/LP detection. According to clinical guidelines, anti-SLA/LP antibodies should be detected using molecular assays, such as ELISA and/or Western blotting (8, 9, 15, 16). LMA is frequently observed in chronic active liver diseases, characterized by linear or membranous fluorescence on the hepatocyte membrane and reticular fluorescence on liver substrate (13, 14). LSP, a liver-specific antibody, exhibits granular cytoplasmic fluorescence on monkey liver substrate but demonstrates minimal fluorescence on rat hepatocytes. Although LSP demonstrates organ specificity, it lacks disease specificity (13, 14).

### 3.6 Anti-mitochondrial antibody

AMA represents the characteristic autoantibody for PBC (8–10). Rat kidney tissue serves as the standard substrate for AMA detection (8, 9, 15, 16, 38, 39). When tested on rat kidney substrate, AMA exhibits cytoplasmic speckled fluorescence, with patterns varying by subtypes (35, 38, 39). AMA-M2 subtype, is observed in both proximal and distal renal tubular epithelial cells, while glomeruli demonstrate weak fluorescence (35, 38, 39). AMA-M6 and AMA-M9 subtypes are restricted to proximal renal tubular epithelial cells (35, 38, 39). AMA-M7 and AMA-M8 subtypes are limited to distal renal tubular epithelial cells (35, 38, 39).

Auxiliary detection of AMA can be performed through analysis of fluorescence patterns on HEp-2 cells, as well as rodent liver and stomach substrates (8, 9, 15, 16, 28, 30, 38). On HEp-2 cells, AMA displays a cytoplasmic reticular pattern (AC-21). On rat liver substrate, AMA induces cytoplasmic speckled fluorescence on hepatocytes. On rat stomach substrate, AMA produces distinct fluorescent staining of parietal cells, whereas chief cells exhibit weaker fluorescence. Additionally, on monkey myocardial substrate, AMA elicits granular fluorescence.

AMA must be distinguished from the fluorescence pattern produced by anti-parietal cell antibodies (PCA). PCA elicits distinct fluorescent staining exclusively on the gastric parietal cells (with weaker fluorescence on chief cells); notably, PCA does not induce cytoplasmic speckled fluorescence on HEp-2 cells, kidney substrates, or liver substrates (40, 41).

AMA is identified based on the reticular/mitochondrial pattern (AC-21) on HEp-2 cells and cytoplasmic speckled fluorescence on proximal and distal renal tubules of rodent kidney substrates.

## 4 Fluorescent patterns of autoantibodies associated with gastrointestinal autoimmune diseases

Gastrointestinal autoimmune diseases primarily encompass autoimmune gastritis (AIG) and autoimmune enteropathy (AIE) (42). Autoantibodies associated with AIG mainly consist of anti-parietal cell antibodies (PCA) and intrinsic factor antibodies (IFA) (40, 41). Both PCA and IFA are commonly detected in the serum of patients with AIG and pernicious anemia (PA) (40, 41, 43). For their detection, ELISA or CLIA is recommended (41, 43). Although the ELISA method is shown to be more sensitive and specific (40, 44), and IIF is no longer widely employed in clinical practice for PCA detection, but a specific explanation is provided herein since PCA is frequently confused with AMA and heterophile antibodies (HA).

Autoantibodies commonly associated with autoimmune enteropathy include anti-goblet cell antibodies (GCA), anti-pancreatic exocrine acinar antibodies (PAB), anti-Saccharomyces cerevisiae antibodies (ASCA), anti-neutrophil cytoplasmic antibodies (ANCA), anti-endomysial antibodies (EMA), anti-reticulin antibodies (ARA), anti-tissue transglutaminase antibodies (anti-tTG), and anti-gliadin antibodies (AGA) (45–48). The combined detection of GCA, PAB, ASCA, and ANCA can assist in differentiating between ulcerative colitis (UC) and Crohn’s disease (CD) (45, 47–50). EMA, ARA, anti-tTG antibodies, and AGA are all associated with celiac disease (46, 51, 52). For the detection of anti-tTG antibodies and AGA, ELISA or CLIA is recommended (46, 51, 52). In contrast, IIF is recommended for the screening of GCA, PAB, ASCA, ANCA, EMA, and ARA (45–52). The morphological characteristics and clinical significance of autoantibodies associated with gastrointestinal autoimmune diseases are summarized in **Table 2**.

**Table 2:** The morphological characteristics and clinical significance of autoantibodies associated with gastrointestinal autoimmune diseases.

The type of autoantibodies	Morphological characteristics	Related autoimmune diseases of the digestive system	Ref.
GCA	The cytoplasm of goblet cells in the small intestinal epithelium demonstrates uniform, diffuse, and poorly demarcated cloud-like fluorescence (monkey intestinal substrate).	Ulcerative colitis (UC).	(48, 49, 56, 57)
PAB	The cytoplasm of exocrine pancreatic cells exhibits various fluorescent staining patterns, including fine speckled, droplet-like, and reticular fluorescence (monkey pancreatic substrate).	Crohn’s disease (CD).	(47–49, 56, 58)
ASCA	The staining of yeast cells and enhanced fluorescence on the cell wall (Saccharomyces cerevisiae smears).	CD.	(47–49, 56, 60)
ANCA	C-ANCA exhibits granular fluorescence uniformly distributed throughout the neutrophil cytoplasm. p-ANCA displays band-like, smooth perinuclear fluorescence surrounding the neutrophil nucleus.	Assist in differentiating between UC and CD.	(8, 9, 15, 16, 47–52)
EMA	The staining surrounding the smooth muscle cells in the muscularis mucosae and muscularis propria of tesophagus, stomach and intestines.	Celiac disease.	(46, 63, 64)

GCA, anti-intestinal goblet cell antibody; PAB, anti-exocrine panlrease antibody; ASCA, anti-saccharomyces cerevisiae antibody; ANCA, anti-neutrophil cytoplasmic antibody; EMA, anti-endomysial antibody.

### 4.1 Anti-parietal cell antibodies

PCA produces intense fluorescent staining of parietal cells on monkey or rodent stomach substrates (40, 41, 43, 44). Using rat stomach substrate as an example, PCA induces significant fluorescent staining of parietal cells (40, 41, 43, 44). Notably, PCA does not trigger cytoplasmic speckled fluorescence on liver substrates, kidney substrates, or HEp-2 cells.

Heterophile antibodies (HA) induce speckled fluorescence on gastric parietal cells and positive staining of the brush border in proximal renal tubular epithelium (53). No specific fluorescence was observed in other substrates. Since HA cross-reacts with PCA, comprehensive assessment based on fluorescent patterns of both gastric tissue and kidney substrates is essential when differentiating between these antibodies using IIF. Both HA and anti-brush border antibodies demonstrate positive staining of the brush border and proximal renal tubular epithelial cells of rat kidney, as well as parietal cells of rat stomach substrate (53–55). However, their target antigens remain unclear, necessitating further investigation to determine whether they represent identical or distinct antibodies. AMA induces significant fluorescent staining of gastric parietal cells and additionally exhibits cytoplasmic speckled fluorescence on kidney substrates.

### 4.2 Anti-intestinal goblet cell antibodies, anti-exocrine panlrease antibodies, anti-saccharomyces cerevisiae antibodies, and anti-neutrophil cytoplasmic antibodies

GCA are primarily detected in patients with ulcerative colitis, while typically being negative in patients with Crohn’s disease and other enteropathies (48, 49). Currently, IIF remains the most commonly employed method for GCA detection (48, 49, 56). The substrate for GCA detection typically consists of frozen sections of primate (e.g., monkey) intestinal tissue, on which the cytoplasm of goblet cells in the small intestinal epithelium demonstrates uniform, diffuse, and poorly demarcated cloud-like fluorescence (57).

PAB are predominantly detected in patients with Crohn’s disease, with IIF being commonly employed for their detection (47–49, 56). The substrate for detecting these antibodies typically consists of frozen sections of primate (e.g., monkey) pancreatic tissue. On this substrate, the cytoplasm of exocrine pancreatic cells exhibits various fluorescent staining patterns, including fine speckled, droplet-like, and reticular fluorescence (58).

ASCA are specific for Crohn’s disease (47–49, 56). Although ELISA is currently recommended for ASCA detection (59), IIF detection offers simplicity and convenience, leading to its widespread clinical application. The substrate for ASCA detection consists of Saccharomyces cerevisiae smears, with positive results characterized by distinct fluorescent staining of yeast cells and enhanced fluorescence on the cell wall (60). This fluorescent pattern resembles that observed in nuclear membrane-type antinuclear antibodies.

ANCA serves as a crucial diagnostic marker for small vessel vasculitis (61), and represents a key autoantibody for differentiating autoimmune enteropathies, including ulcerative colitis and Crohn’s disease (47–52). International consensus guidelines recommend using IIF with ethanol-fixed and formaldehyde-fixed neutrophils as standard substrates, followed by ELISA for target antigen confirmation (8, 9, 15, 16, 50).

ANCA encompasses a group of autoantibodies targeting various components within human neutrophil cytoplasm. Based on their target antigens, ANCA is classified into cytoplasmic ANCA (C-ANCA) and perinuclear ANCA (P-ANCA) (8, 9, 15, 16, 50). On formaldehyde-fixed neutrophil (HCHO) substrates, both C-ANCA and P-ANCA demonstrate granular fluorescence uniformly distributed throughout the neutrophil cytoplasm (8, 9, 15, 16, 50). The key distinction between these antibodies lies in their fluorescence patterns on ethanol-fixed neutrophil (EOH) substrates. C-ANCA exhibits granular fluorescence uniformly distributed throughout the neutrophil cytoplasm with no nuclear fluorescence (8, 9, 15, 16, 50). In contrast, p-ANCA displays band-like, smooth perinuclear fluorescence surrounding the neutrophil nucleus (8, 9, 15, 16, 50). The target antigen corresponding to C-ANCA is Proteinase 3 (PR3) (61, 62), whereas the target antigen for P-ANCA is myeloperoxidase (MPO), followed by elastase (EL), cathepsin G (CG), and other antigens (61, 62). When determining the ANCA type, interference from antinuclear antibodies (ANA) must be excluded.

### 4.3 Anti-endomysial antibodies and anti-reticulin antibodies

Both EMA and ARA are specific for celiac disease (46, 63, 64). EMA can be detected using primate (e.g., monkey) esophageal, gastric, and intestinal tissue substrates. For EMA detection, IIF is recommended, with monkey liver serving as the most optimal substrate, while the esophagus and small intestine serving as alternative standard substrates (46, 63, 64). EMA positivity is characterized by reticular fiber-like fluorescent staining surrounding the smooth muscle cells in the muscularis mucosae and muscularis propria of esophagus, stomach and intestine (23). Additionally, continuous linear fluorescence around hepatic sinusoids on liver tissue substrates can provide diagnostic support. EMA can be classified into two subtypes: IgA-EMA and IgG-EMA (65). IIF for EMA detection is not the first choice for routine screening; instead, it is primarily employed for further diagnostic evaluation when tissue transglutaminase-IgA (tTG-IgA) test results are weakly positive.

On frozen sections of gastric tissue substrate, ARA-induced fluorescence demonstrates inter-glandular fiber-like staining in the mucosa and a “honeycomb-like” fluorescent pattern in the muscularis propria (23, 66). Additionally, fluorescent staining surrounding blood vessels, renal tubules and glomeruli can be observed on rodent (e.g., rat) kidney substrate. On liver tissue substrate, three distinct fluorescence patterns can be identified: the nodular fluorescence on connective tissue in the portal triad, the filamentous fluorescence extending from hepatic sinusoids into the liver parenchyma, and “hair-like” fluorescence within the liver parenchyma (23, 66). No characteristic patterns have been identified on other tissue substrates. The fluorescence pattern of ARA resembles those of ASMA and elastin. Careful differentiation is essential, with specific distinctions detailed in the previous section (2.1).

## 5 Discussion

Autoantibody detection is a critical component of laboratory testing for digestive system autoimmune diseases. For the majority of autoantibody assays, the optimal diagnostic workflow consists of initial screening via IIF, followed by confirmatory testing of target antibodies using qualitative or semiquantitative secondary platforms such as ELISA, immunoblotting, and radioimmunoassay (RIA) (8, 9, 15, 16). Notably, RIA requires stringent handling precautions and should only be performed in laboratories certified for radioactive isotope manipulation. After delineating the patient’s specific autoantibody profile, serial quantification of antibodies can be conducted using CLIA or ELISA. This tiered testing algorithm enables clinicians to facilitate disease diagnosis, evaluate therapeutic response, and predict clinical prognosis. Currently, quantitative detection methods (including CLIA, ELISA, etc) are convenient to perform, widely applied, and highly specific; nevertheless, their sensitivity is in some cases lower than that of IIF (67). Relevant guidelines recommend the use of CLIA or ELISA for detecting antibodies such as PCA and IFA (41, 43). While IIF is technically demanding and has lower specificity, it is more sensitive than quantitative assays under certain conditions. According to current clinical guidelines, IIF should be used as a first-line screening method for most autoantibodies (8, 9, 15, 16). Furthermore, numerous as-yet unidentified antibody targets can only be revealed through fluorescence-based detection. In addition, implementation of detection platforms such as CLIA and immunoblotting requires dedicated equipment, and their high cost limits their accessibility in many primary care hospitals. Despite being significant time expenditure, demand for specialized expertise, and the deficit of standardization, the IAIHG (International Autoimmune Hepatitis Groups) Autoimmune Serology Committee still advocate IIF as the preferred, or sometimes the exclusive diagnostic option, grounding in an assessment of the methodology’s economic practicality, diagnostic import, and overall viability for most healthcare institutions.

Currently, research on the automatic fluorescence interpretation of autoantibodies mainly focuses on AI model studies based on cell morphology for ANA, ANCA, and dsDNA antibodies (68, 69). Research on the automatic recognition of corresponding autoantibodies (such as ASMA and AMA) on tissue substrates is still in its early stages. Although automatic image acquisition systems have been developed, mature models for automatically identifying antibody types from tissue substrates have not yet been established. The reasons for this are that the fluorescence patterns on tissue substrates vary significantly, involve a wide range of substrate types, and the interpretation of autoantibodies on these substrates is highly challenging. Considering the difficulty of interpreting IIF when detecting related autoantibodies, this article will summarize the staining patterns of various digestive system autoantibodies detected by IIF based on tissue structure, and differentiate them from other abnormal fluorescent staining that may be easily confused in clinical practice. We hope this can effectively assist fluorescence interpretation experts in more accurately identifying relevant autoantibodies, guiding clinical diagnosis more precisely, and better assessing therapeutic efficacy and prognosis. In addition, it can better facilitate research on automatic interpretation models for tissue substrates. By developing an in-depth, granular understanding of the identification criteria for autoantibody fluorescence images, we can lay a more robust theoretical foundation to boost the diagnostic accuracy of AI-based automated interpretation models under development. Analogous to well-established automated interpretation frameworks like ANA-AI currently implemented for routine clinical testing, automated models with tissue substrates for autoantibody interpretation are expected to be developed to substantially cut laboratory technicians’ workload. Nevertheless, owing to the stringent specificity standards governing medical documentation, every AI-generated report requires validation by qualified clinicians before issuance. Accordingly, expert physicians remain irreplaceable for IIF result assessment, and AI systems merely function as auxiliary support.

This review aims to summarize and classify the fluorescent patterns of autoantibodies associated with autoimmune digestive diseases and their clinical significance, focusing on the differentiation of overlapping fluorescent patterns on IIF. Currently, autoimmune hepatitis-associated autoantibodies that still rely on IIF include ASMA, ANA, LKM, LC-1, LMA, LSP, and AMA. Due to interference from ARA and elastin, the immunofluorescence patterns of ASMA are the most challenging to interpret. By analyzing their differences in tissue localization, we carefully distinguished ASMA, elastin, and ARA. ASMA will stain the interglandular contractile fibers within the mucosa lamina propria, the muscularis mucosa and the muscularis propria of the stomach tissue as well as the muscle layer of the arterioles that may be present in any of the tissue sections. ARA will stain peritubular fibers, vascular endothelium and perivascular fibers. Anti-elastin antibodies mainly stain ligaments and blood vessel walls. These findings not only help laboratory diagnosticians better identify ASMA and avoid misdiagnosis caused by elastin and ARA, but also clarify the distinction between elastin and ARA. Such accurate identification is crucial for the diagnosis of AIH and celiac disease. AMA, PCA, and HA all produce prominent fluorescence on gastric parietal cells. AMA exhibits granular cytoplasmic fluorescence on other substrates, whereas PCA staining is completely negative. HA shows marked fluorescence on the brush border of renal tissue, but no distinct specific fluorescence is observed on other substrates. Similarly, more accurate differentiation of AMA, PCA, and HA provides important clues for the differential diagnosis of PBC from autoimmune gastritis. In particular, whether HA and anti-brush border antibodies are identical and their clinical significance remains unclear; more precise identification of the actual antibodies is of practical significance for further exploring their clinical value. At present, autoantibodies associated with autoimmune bowel diseases that are detectable by IIF include GCA, PAB, ASCA, ANCA, EMA, and ARA. Among these, identification of ANCA is relatively challenging and requires exclusion of interference from certain antinuclear antibodies. Of note, ANCA is not a specific antibody for autoimmune bowel disease and is used in combination with other autoantibodies for its diagnosis.

Fluorescence intensity is quantified by serial dilution titers. Clinically, we observed that healthy adults from different geographic regions often exhibit low-titer antinuclear antibodies to varying extents, a trend also seen with other autoantibodies. Consequently, it is recommended that laboratories establish region-specific positivity thresholds based on local population demographics. In the context of diagnosing autoimmune liver diseases in pediatric patients, lower autoantibody titers are considered clinically significant. Therefore, establishing distinct cutoff values for children and adults is advisable when defining positivity for various autoantibodies.

This review only summarizes the identification of fluorescence patterns and the clinical significance of autoantibodies associated with digestive system autoimmune diseases that require IIF detection, and does not cover related autoantibodies measurable by other assay methods. Given the limited spectrum of antibody types discussed, its value in aiding the diagnosis, treatment, and prognosis evaluation of digestive system autoimmune diseases is relatively restricted. In addition, during the analysis process, we focused primarily on the interpretation of fluorescence patterns, which led to insufficient discussion of diagnostic performance and comparison with other methodologies. Consequently, this analysis is somewhat incomplete and one−sided, and its clinical applicability is therefore limited.

IIF serves as a powerful diagnostic adjunct. Despite the challenges including operational complexity, result accuracy, and result interpretation, improving the accuracy of IIF results remains an essential endeavor that every laboratory professional must undertake.

## References

[1] Fan S Prüss H . Pathogenic mechanisms of autoantibodies in neurological autoimmune diseases. Curr Opin Immunol. (2025) 95:102594. doi: 10.1016/j.coi.2025.102594 40609230

[2] Pérez-Bucio C Behere A Landegren N . Mechanisms of autoimmune-mediated paraneoplastic syndromes: immune tolerance and disease pathogenesis. Front Immunol. (2025) 16:1608934. doi: 10.3389/fimmu.2025.1608934 40416967 PMC12098635

[3] Xiao Z Miller J Zheng S . An updated advance of autoantibodies in autoimmune diseases. Autoimmun Rev. (2021) 20:102743. doi: 10.1016/j.autrev.2020.102743 33333232

[4] Xu H Li S Liu S Zuo Y . A conceptual review of gut, skin, and oral microbiota in autoimmune bullous diseases: from dysbiosis to therapeutic potential. J Inflammation Res. (2025) 18:13925–43. doi: 10.2147/jir.s551394 41080149 PMC12515014

[5] Shang Y Han Y . Autoimmune diseases of digestive system: challenges and opportunities. Zhonghua Nei Ke Za Zhi. (2025) 64:183–5. doi: 10.3760/cma.j.cn112138-20250122-00055 40050083

[6] Gleeson D Bornand R Brownlee A Dhaliwal H Dyson J Hails J . British Society of Gastroenterology guidelines for diagnosis and management of autoimmune hepatitis. Gut. (2025) 74:1364–409. doi: 10.1136/gutjnl-2024-333171 40169244 PMC12421125

[7] Rossi C Lenti M Merli S Santacroce G Di Sabatino A . Allergic manifestations in autoimmune gastrointestinal disorders. Autoimmun Rev. (2022) 21:102958. doi: 10.1016/j.autrev.2021.102958 34560305

[8] Terziroli Beretta-Piccoli B Mieli-Vergani G Vergani D . Serology in autoimmune hepatitis: a clinical-practice approach. Eur J Internal Med. (2018) 48:35–43. doi: 10.1016/j.ejim.2017.10.006 29056396

[9] Dalekos G Gatselis N . Autoimmune serology testing in clinical practice: an updated roadmap for the diagnosis of autoimmune hepatitis. Eur J Internal Med. (2023) 108:9–17. doi: 10.1016/j.ejim.2022.11.013 36400668

[10] Shah S Bowlus C . Autoimmune markers in primary biliary cholangitis. Clinics Liver Dis. (2024) 28:93–101. doi: 10.1016/j.cld.2023.07.002 37945165

[11] Lapierre P Alvarez F . Type 2 autoimmune hepatitis: genetic susceptibility. Front Immunol. (2022) 13:1025343. doi: 10.3389/fimmu.2022.1025343 36248826 PMC9556705

[12] Brahim I Brahim I Hazime R Admou B . Autoimmune hepatitis: immunological diagnosis. Presse Med (Paris France 1983). (2017) 46:1008–19. doi: 10.1016/j.lpm.2017.08.012 28919271

[13] Meliconi R Facchini A Miglio F Trevisan A Alberti A Realdi G . Liver-specific autoantibodies in chronic hepatitis B virus infection. Clin Sci (London Engl 1979). (1985) 68:123–6. doi: 10.1042/cs0680123 3967461

[14] Nagai S Manns M Meyer zum Büschenfelde K . Liver membrane autoantibodies: methods for their detection and clinical significance. Tokai J Exp Clin Med. (1982) 7:145–58. 7048633

[15] Dalekos G Gatselis N Drenth JP Heneghan M Jørgensen M Lohse AW . EASL Clinical Practice Guidelines on the management of autoimmune hepatitis. J Hepatol. (2025) 83:453–501. doi: 10.1016/j.jhep.2025.03.017 40348684

[16] Dalekos G Papatheodoridis G Koskinas J Goulis I Rigopoulou E Tiniakos D . Hellenic Association for the Study of the Liver (HASL): revised clinical practice guidelines for autoimmune hepatitis. Ann Gastroenterol. (2024) 37:623–54. doi: 10.20524/aog.2024.0924 39568707 PMC11574148

[17] Granito A Muratori L Muratori P Pappas G Guidi M Cassani F . Antibodies to filamentous actin (F-actin) in type 1 autoimmune hepatitis. J Clin Pathol. (2006) 59:280–4. doi: 10.1136/jcp.2005.027367 16505279 PMC1860354

[18] Chretien-Leprince P Ballot E Andre C Olsson N Fabien N Escande A . Diagnostic value of anti-F-actin antibodies in a French multicenter study. Ann N Y Acad Sci. (2005) 1050:266–73. doi: 10.1196/annals.1313.028 16014542

[19] Halper J . Basic components of connective tissues and extracellular matrix: fibronectin, fibrinogen, laminin, elastin, fibrillins, fibulins, matrilins, tenascins and thrombospondins. Adv Exp Med Biol. (2021) 1348:105–26. doi: 10.1007/978-3-030-80614-9_4 34807416

[20] Ruiz T Ferrato L de Souza L Brito-Filho G Leonel E Taboga S . The elastic system: a review of elastin-related techniques and hematoxylin-eosin/phloxine applicability for normal and pathological tissue description. Acta Histochem. (2024) 126:152209. doi: 10.1016/j.acthis.2024.152209 39442433

[21] Kárpáti S Stolz W Meurer M Krieg T Braun-Falco O . Extracellular binding sites of IgA anti-jejunal antibodies on normal small bowel detected by indirect immunoelectronmicroscopy. J Invest Dermatol. (1991) 96:228–33. doi: 10.1111/1523-1747 1899445

[22] Nasr M Ali Deeb A Badra G El Sayed I . Lack of any relationship between circulating autoantibodies and interleukin–6 levels in Egyptian patients infected with the hepatitis C virus. Asian Pacific J Cancer Prev APJCP. (2016) 17:4977–9. doi: 10.22034/APJCP.2016.17.11.4977 PMC5454706 28032726

[23] Lock R Gilmour J Unsworth D . Anti-tissue transglutaminase, anti-endomysium and anti-R1-reticulin autoantibodies-the antibody trinity of coeliac disease. Clin Exp Immunol. (1999) 116:258–62. doi: 10.1046/j.1365-2249.1999.00909.x 10337016 PMC1905294

[24] Murota M Nishioka M Fujita J Dobashi N Wu F Ohtsuki Y . Anti-cytokeratin antibodies in sera of the patients with autoimmune hepatitis. Clin Exp Immunol. (2001) 125:291–9. doi: 10.1046/j.1365-2249.2001.01568.x 11529922 PMC1906119

[25] de Melo Cruvinel W Ferreira G de Souza L da Costa Veloso Neto W Gomes C Francescantonio P . The spectrum of nuclear patterns with stained metaphase chromosome plate: morphology nuances, immunological associations, and clinical relevance. Clin Chem Lab Med. (2025) 63:1915–27. doi: 10.1515/cclm-2025-0286 40526276

[26] Satoh M Ceribelli A Hasegawa T Tanaka S . Clinical significance of antinucleolar antibodies: biomarkers for autoimmune diseases, Malignancies, and others. Clin Rev Allergy Immunol. (2022) 63:210–39. doi: 10.1007/s12016-022-08931-3 35258843

[27] Damoiseaux J Andrade L Carballo O Conrad K Francescantonio P Fritzler M . Clinical relevance of HEp-2 indirect immunofluorescent patterns: the International Consensus on ANA patterns (ICAP) perspective. Ann Rheumatic Dis. (2019) 78:879–89. doi: 10.1136/annrheumdis-2018-214436 30862649 PMC6585284

[28] Wei Q Jiang Y Xie J Yang M Zhang Y Wu Z . Investigation and analysis of HEp 2 indirect immunofluorescence titers and patterns in various liver diseases. Clin Rheumatol. (2020) 39:2425–32. doi: 10.1007/s10067-020-04950-7 32103375

[29] Andrade L Klotz W Herold M Musset L Damoiseaux J Infantino M . Reflecting on a decade of the international consensus on ANA patterns (ICAP): accomplishments and challenges from the perspective of the 7th ICAP workshop. Autoimmun Rev. (2024) 23:103608. doi: 10.1016/j.autrev.2024.103608 39187221

[30] Terziroli Beretta-Piccoli B Mieli-Vergani G Vergani D . The clinical usage and definition of autoantibodies in immune-mediated liver disease: a comprehensive overview. J Autoimmun. (2018) 95:144–58. doi: 10.1016/j.jaut.2018.10.004 30366656

[31] Fabien N Desbos A Bienvenu J Magdalou J . Autoantibodies directed against the UDP-glucuronosyltransferases in human autoimmune hepatitis. Autoimmun Rev. (2004) 3:1–9. doi: 10.1016/s1568-9972(03)00051-x 14871643

[32] López-García M Dansette P Coloma J . Kinetics of tienilic acid bioactivation and functional generation of drug-protein adducts in intact rat hepatocytes. Biochem Pharmacol. (2005) 70:1870–82. doi: 10.1016/j.bcp.2005.09.026 16257391

[33] Bonierbale E Valadon P Pons C Desfosses B Dansette P Mansuy D . Opposite behaviors of reactive metabolites of tienilic acid and its isomer toward liver proteins: use of specific anti-tienilic acid-protein adduct antibodies and the possible relationship with different hepatotoxic effects of the two compounds. Chem Res Toxicol. (1999) 12:286–96. doi: 10.1021/tx980136z 10077492

[34] Larrey D . Drug-induced hepatitis: epidemiologic, clinical, diagnostic and physiopathologic aspects in 1995. La Rev Med Interne. (1995) 16:752–8. doi: 10.1016/0248-8663(96)80784-3 8525155

[35] Liberal R Grant C Longhi M Mieli-Vergani G Vergani D . Diagnostic criteria of autoimmune hepatitis. Autoimmun Rev. (2014) 13:435–40. doi: 10.1007/978-3-030-51709-0_26 24418295

[36] Villalta D Mytilinaiou M Elsner M Hentschel C Cuccato J Somma V . Autoantibodies to asialoglycoprotein receptor (ASGPR) in patients with autoimmune liver diseases. Clin Chim Acta; Int J Clin Chem. (2015) 450:1–5. doi: 10.1016/j.cca.2015.07.021 26220739

[37] Toh B . Diagnostic autoantibodies for autoimmune liver diseases. Clin Trans Immunol. (2017) 6:e139. doi: 10.1038/cti.2017.14 28690845 PMC5493583

[38] Bogdanos D Komorowski L . Disease-specific autoantibodies in primary biliary cirrhosis. Clin Chim Acta; Int J Clin Chem. (2011) 412:502–12. doi: 10.1016/j.cca.2010.12.019 21185272

[39] Berg P Klein R . Mitochondrial antigens and autoantibodies: from anti-M1 to anti-M9. Klinische Wochenschrift. (1986) 64:897–909. doi: 10.1007/bf01728613 3537495

[40] Shah S Piazuelo M Kuipers E Li D . AGA Clinical Practice Update on the diagnosis and management of atrophic gastritis: expert review. Gastroenterology. (2021) 161:1325–32.e7. doi: 10.1053/j.gastro.2021.06.078 34454714 PMC8740554

[41] Khan S Del-Duca C Fenton E Holding S Hirst J Doré P . Limited value of testing for intrinsic factor antibodies with negative gastric parietal cell antibodies in pernicious anaemia. J Clin Pathol. (2009) 62:439–41. doi: 10.1136/jcp.2008.060509 19398595

[42] Garabatos N Angelats E Santamaria P . Mechanistic and therapeutic advances in immune-mediated gastrointestinal disorders. J Allergy Clin Immunol. (2025) 156(5):1133–1159. doi: 10.1016/j.jaci.2025.07.024 40783002

[43] Rusak E Chobot A Krzywicka A Wenzlau J . Anti-parietal cell antibodies - diagnostic significance. Adv Med Sci. (2016) 61:175–9. doi: 10.1016/j.advms.2015.12.004 26918709

[44] Bizzaro N Antico A . Diagnosis and classification of pernicious anemia. Autoimmun Rev. (2014) 13:565–8. doi: 10.1016/j.autrev.2014.01.042 24424200

[45] Diez-Martin E Hernandez-Suarez L Muñoz-Villafranca C Martin-Souto L Astigarraga E Ramirez-Garcia A . Inflammatory bowel disease: a comprehensive analysis of molecular bases, predictive biomarkers, diagnostic methods, and therapeutic options. Int J Mol Sci. (2024) 25:7062. doi: 10.3390/ijms25137062 39000169 PMC11241012

[46] Gandini A Gededzha M De Maayer T Barrow P Mayne E . Diagnosing coeliac disease: a literature review. Hum Immunol. (2021) 82:930–6. doi: 10.1016/j.humimm.2021.07.015 34462157

[47] Conrad K Roggenbuck D Laass M . Diagnosis and classification of ulcerative colitis. Autoimmun Rev. (2014) 13:463–6. doi: 10.1016/j.autrev.2014.01.028 24424198

[48] Laass M Roggenbuck D Conrad K . Diagnosis and classification of Crohn's disease. Autoimmun Rev. (2014) 13:467–71. doi: 10.1016/j.autrev.2014.01.029 24424189

[49] Bernstein C Eliakim A Fedail S Fried M Gearry R Goh K . World gastroenterology organisation global guidelines inflammatory bowel disease: update august 2015. J Clin Gastroenterol. (2016) 50:803–18. doi: 10.1097/mcg.0000000000000660 27741097

[50] Sepiashvili L Kenyon S . Clinical, methodological, and practical considerations for algorithmic testing in autoimmune serology. J Appl Lab Med. (2022) 7:268–80. doi: 10.1093/jalm/jfab121 34996074

[51] Tonutti E Bizzaro N . Diagnosis and classification of celiac disease and gluten sensitivity. Autoimmun Rev. (2014) 13:472–6. doi: 10.1016/j.autrev.2014.01.043 24440147

[52] Husby S Koletzko S Korponay-Szabó IR Mearin ML Phillips A Shamir R . European Society for Pediatric Gastroenterology, Hepatology, and Nutrition guidelines for the diagnosis of coeliac disease. J Pediatr Gastroenterol Nutr. (2012) 54:136–60. doi: 10.1097/mpg.0b013e31821a23d0 22197856

[53] McDonald BL Hawkins BR Dawkins RL Davey MG . Characterization of human heterophile antibodies apparently induced by alloimmunization. Vox Sang. (1977) 33:143–9. doi: 10.1159/000467503 331681

[54] Skogh T Heuman R Tagesson C . Anti-brush border antibodies (ABBA) in Crohn's disease. J Clin Lab Immunol. (1982) 9:147–50. 6762441

[55] Haddad A Goldinger JM Van Liew JB Noble B . Kidney immunopathology and pathophysiology in rats immunized with proximal tubule cell brush border or basolateral membrane vesicles. Immunol Invest. (1987) 16:213–25. doi: 10.3109/08820138709030577 3311982

[56] Chen Q Huang S Wu Y Zhang S Liu Q Chen M . Age and gender: Affecting the positive rates of serum PAB and ANCA in patients with inflammatory bowel disease. Gastroenterol Res Pract. (2021) 2021:4963641. doi: 10.1155/2021/4963641 34394344 PMC8360740

[57] Lai Y Ye J Zhang Y Chang H Zhang H Shi X . Adult-onset generalized autoimmune enteropathy involving small intestine and colon: report of a case and review of literature. Zhonghua Bing Li Xue Za Zhi Chin J Pathol. (2015) 44:32–6. doi: 10.3760/cma.j.issn.0529-5807.2015.01.007 25765028

[58] Bogdanos DP Rigopoulou EI Smyk DS Roggenbuck D Reinhold D Forbes A . Diagnostic value, clinical utility and pathogenic significance of reactivity to the molecular targets of Crohn's disease specific-pancreatic autoantibodies. Autoimmun Rev. (2011) 11:143–8. doi: 10.1016/j.autrev.2011.09.004 21983481

[59] Sendid B Cornu M Cordier C Bouckaert J Colombel JF Poulain D . From ASCA breakthrough in Crohn's disease and Candida albicans research to thirty years of investigations about their meaning in human health. Autoimmun Rev. (2024) 23:103486. doi: 10.1016/j.autrev.2023.103486 38040100

[60] Desplat-Jégo S Johanet C Escande A Goetz J Fabien N Olsson N . Update on anti-Saccharomyces cerevisiae antibodies, anti-nuclear associated anti-neutrophil antibodies and antibodies to exocrine pancreas detected by indirect immunofluorescence as biomarkers in chronic inflammatory bowel diseases: results of a multicenter study. World J Gastroenterol. (2007) 13:2312–8. doi: 10.3748/wjg.v13.i16.2312 17511029 PMC4147139

[61] Papadopoulou M Karamanakos A . Points to consider in the management of ANCA-associated vasculitis. Autoimmun Rev. (2025) 24:103894. doi: 10.1016/j.autrev.2025.103894 40754136

[62] Guchelaar NAD Waling MM Adhin AA van Daele PLA Schreurs MWJ Rombach SM . The value of anti-neutrophil cytoplasmic antibodies (ANCA) testing for the diagnosis of ANCA-associated vasculitis, a systematic review and meta-analysis. Autoimmun Rev. (2021) 20:102716. doi: 10.1016/j.autrev.2020.102716 33197574

[63] Bonaci-Nikolic B Andrejevic S Radlovic N Davidovic I Sofronic L Spuran M . Serological and clinical comparison of children and adults with anti-endomysial antibodies. J ClinImmunol. (2007) 27:163–71. doi: 10.1007/s10875-006-9062-y 17243009

[64] Shiha MG Chetcuti Zammit S Elli L Sanders DS Sidhu R . Updates in the diagnosis and management of coeliac disease. Best Pract Res Clin Gastroenterol. (2023) 64-65:101843. doi: 10.1016/j.bpg.2023.101843 37652646

[65] Cataldo F Lio D Marino V Picarelli A Ventura A Corazza GR . IgG(1) antiendomysium and IgG antitissue transglutaminase (anti-tTG) antibodies in coeliac patients with selective IgA deficiency. Working Groups on Celiac Disease of SIGEP and Club del Tenue. Gut. (2000) 47:366–9. doi: 10.1136/gut.47.3.366 10940273 PMC1728054

[66] Boige V Bouhnik Y Delchier JC Jian R Matuchansky C André C . Anti-endomysium and anti-reticulin antibodies in adults with celiac disease followed-up in the Paris area. Gastroenterol Clin Biol. (1996) 20:931–7. 9119181

[67] El-Chennawi FA Mosaad YM Habib HM El-Degheidi T . Comparative study of antinuclear antibody detection by indirect immunofluorescence and enzyme immunoassay in lupus patients. Immunol Invest. (2009) 38:839–50. doi: 10.3109/08820130903278097 19860592

[68] Durmuş MA Kömeç S . Comparison of artificial intelligence applications and commercial system performances using selected ANA IIF images. Immunol Res. (2025) 73:70. doi: 10.1007/s12026-025-09623-8 40227504

[69] Bertin D Bongrand P Bardin N . Comparison of the capacity of several machine learning tools to assist immunofluorescence-based detection of anti-neutrophil cytoplasmic antibodies. Int J Mol Sci. (2024) 25:3270. doi: 10.1101/2024.01.26.24301725 38542244 PMC10969855

